# The battle against Covid-19: the experience of an Egyptian radiology department in a university setting

**DOI:** 10.1186/s43055-020-00335-7

**Published:** 2020-10-28

**Authors:** Shaimaa AbdelSattar Mohammad, Ahmed M. Osman, Abeer Maghawry Abd-Elhameed, Khaled A. Ahmed, Noha M. Taha, Ayman Saleh, Ashraf Omar, Mahmoud El-Meteini, Mona Adel Mohamed

**Affiliations:** 1 Department of Radiodiagnosis, Faculty of Medicine, Ain Shams University, 9-Lotfi Elsayed St. University Staff buildings, Abbasya, Cairo, Egypt; 2 Department of Cardiology, Faculty of Medicine, Ain Shams University, Cairo, Egypt; 3 Department of General Surgery, Faculty of Medicine, Ain Shams University, Cairo, Egypt

**Keywords:** COVID-19, Pandemic, Preparedness, education, Radiology department, Hospital, University Administration

## Abstract

**Background:**

The current COVID-19 pandemic has resulted in marked and rapid changes to the standing policies of radiology departments globally. The aim of this review article is to describe the various processes implemented by a radiology department in an educational institution in a resource limited country during the COVID-19 crisis, giving insights into the adopted strategies in other institutions in developed countries.

**Main body:**

Our preparedness strategy was directed into five main domains: protection and wellness of radiology faculty and staff, radiological examinations and patients’ safety, education, research, and financial support. By implementing new strategies, we found that work reorganization through the use of home PACS provided safe and effective reporting service, low infection rate with zero mortality, and online lectures and theses defense were successful. Furthermore, governmental support and donations were helpful in facing financial challenges during the pandemic. A comprehensive literature review search for policies adopted by other radiology departments in the world was performed. The adopted strategies of various centers are generally similar to ours aiming for mitigating the spread of the virus, keeping good patients’ care, and maintaining the educational process. Few policy differences across institutions were found in the reporting strategy of COVID-19 pneumonia and according to the availability of resources.

**Conclusion:**

Covid-19 pandemic has opened the door for changes in the radiology department policies with renewed focus on educational, clinical, and scientific strategies. Documentation of the dynamic modifications of everyday practices and lessons learned are important as a reference for preparedness for possible second surge or future crisis.

## Background

Since the announcement of the corona virus 19 virus (COVID-19) outbreak in China in late December 2019, the number of cases has increased exponentially worldwide. This viral infection causes respiratory illness that can be severe and deadly in some patients. The virus was first announced in Egypt in March 2020 with rapid increase in the number of cases to date [1].

While the diagnosis of COVID-19 is essentially confirmed by the detection of viral RNA using reverse-transcription polymerase chain reaction (PCR), imaging plays an essential role in screening, triage, and management of patients with concurrent increase in the workload in the radiology department with increasing risk of infection [2]. Practical and efficient measures to control and prevent the spread of infection should be determined and followed [3, 4]. Moreover, the radiology departments in university hospitals not only provide clinical management for many patients but also have educational and research roles. University teachers and researchers face many challenges in the time of pandemic [5, 6]. COVID-19 crisis was a new experience facing our radiology department and institution unveiling challenges, limited resources, and areas of unpreparedness. Documentation of the dynamic modifications of the everyday practices and lessons learned within the radiology department is important for management of current situation and preparedness for possible second surge or crisis. Searching the literature, few recent studies have documented the changes within the radiology departments in institutions in more developed countries in regard to clinical management, education and research [7–9].

Being one of the largest public university hospital in Egypt and located in the heart of its capital, our radiology department not only provides service for millions of patients (COVID-19 and non-COVID-19 patients) but also has an essential role in medical education and research. Our approach to face the challenges of this crisis has been constantly updated since the start of the pandemic in order to follow and implement the government and institutional guidelines for safe and effective management of patients as well as ensuring safety of clinicians, educators, researchers, staff, and students. A consensus report about hospital strategy in the management of COVID-19 cases was recently published [10].

**Fig. 1:**
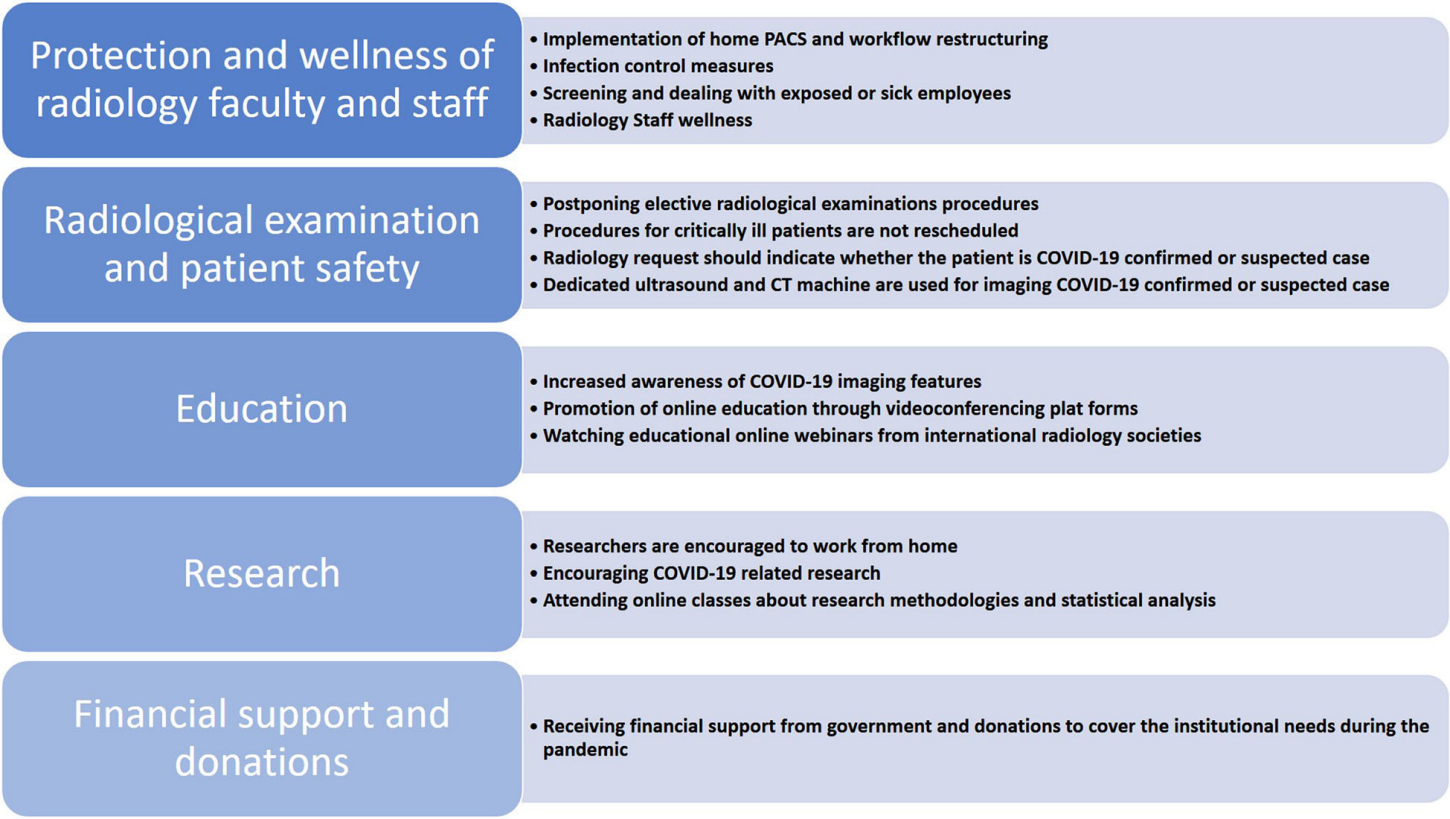
Radiology department preparedness strategy when facing COVID-19 pandemic

The aim of this article is to describe the various processes that have been implemented in an Egyptian academic radiology department during the COVID-19 crisis from the administrative, educational, and research aspects aiming for the lowest risks and best practices. Documentation of our initial response, the constantly changing modifications of our policies and procedures within the radiology department, and the lessons learned are important for preparedness for possible second surge or future crisis. To our knowledge, this is the first COVID 19 radiology department in a university hospital report from an African developing country. Our efforts were directed into five main domains (Fig. 1): (1) *radiology faculty and staff protection and wellness* covering the various processes that have been implemented to protect all personnel in the radiology department and mitigating the spread of COVID-19, and how we could manage to maintain mental and social wellbeing during the crisis; (2) the newly adopted *Radiological examination* adopted to reorganize imaging procedures in order to safely and effectively image COVID and non-COVID patients; (3) *educational* strategies that have been implemented in teaching medical students and postgraduate candidates; (4) the impact on research strategies; and (5) *governmental financial support and donations* that helped face financial challenges during the pandemic.

## Main text

### First domain: protection and wellness of radiology faculty and staff

#### Accessing picture archiving and communication system and workflow restructuring

Before COVID-19 pandemic, our department had implemented home access to picture archiving and communication system (PACS) (Fuji synapse), but with very limited use, as we relied predominantly on physical existence within the hospital. Physical existence was essential for one on one interactions between the radiologists and residents for teaching purposes, also between the referring clinicians and technologists. Accessing PACS was only limited to the on-call shifts. In addition, accessing PACS from home was not a common practice because radiologists could not ensure reliable and stable internet connection, a challenge commonly seen in developing countries.

After COVID-19 pandemic and upon the guidance of the Centers of Disease and Control (CDC), physical distancing is considered an effective way for mitigating the spread of COVID-19 [11]. Non-essential and vulnerable staff with co-morbidities and risk factors were urged to stay at home.

The state and university officials had worked diligently to improve the internet access in the country and across the university. To achieve physical distancing, encouraging the radiologists to access PACS from home for remote reporting and workflow restructuring was the only convenient solution. It not only enabled physical distancing but also ensured interpretation of various radiological examinations in timely manner during the pandemic [5].

Fortunately, due to limited resources and difficulty of purchasing workstations for home PACS, our radiologists updated their personal computers to act as PACS workstations. Similar efforts deploying home PACS in other Radiology departments in the world were also documented. The radiology department at University of Washington School of Medicine and the University of Alabama at Birmingham upgraded their PACS servers and provided more home workstations for radiologists. The University of Alabama at Birmingham primarily provided workstations for emergency, abdominal, cardiothoracic, musculoskeletal, and neuroradiology sections. Nuclear medicine was provided an alternative route enabling them to log into their hospital workstation [12].

Information technology specialists delivered their technical support remotely through TeamViewer Remote Access software. Remote reading of radiology cases had a great advantage in that it opened the opportunity for increased collaborations and easier access of the senior radiologists if a second opinion was needed in difficult cases.

As imaging volume for non-emergency cases was markedly diminished and to promote physical distancing, our daily work routine schedules were restructured for radiologists, residents, and technologists. Only two or three readers (residents and instructor level) were present in person per room with at least 6 feet apart. At least one radiologist should be available on-site to provide residents and technologists with support and reassurance. In addition, an on-call radiology senior faculty was present for 24-h support.

On the contrary, with the increase in the number of thoracic imaging cases over time, and with the construction of 2 new university dedicated quarantine and field hospitals for COVID-19 patients care, the working hours of thoracic imaging team were restructured again for the increased demands for rapid reporting of chest X-rays and CT scans. In addition, more radiologists from other radiology subspecialties were recruited (after being trained on reporting of Chest CT for COVID-19). Moreover, regular on site and virtual multidisciplinary meetings were held to discuss our newly adopted reporting structure, and the radiological and clinical data of admitted COVID-19 cases. Multidisciplinary teams included members from thoracic imaging group, pulmonologists, internal medicine, geriatric medicine, intensive care unit, infection control unit, public health, and community medicine. Additionally, differential diagnosis of the radiological findings was usually discussed with the clinicians and pulmonologists over the phone if more clinical information was needed.

#### Infection control measures

**Fig. 2:**
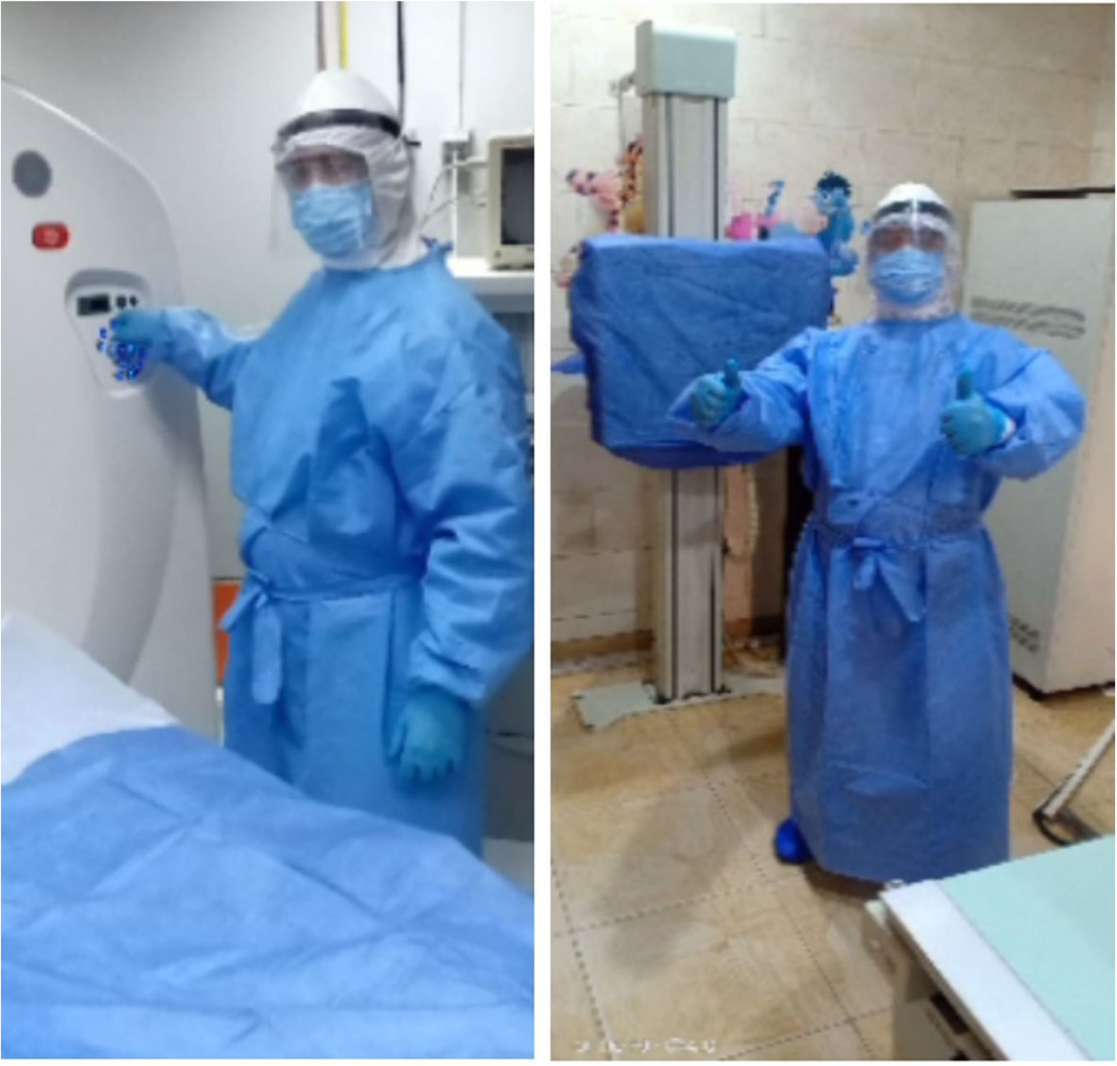
Protection in the imaging rooms: a technologist is wearing PPE and standing beside the CT gantry (**a**); and X-ray bucky (**b**). The Imaging table of the CT scanner and the X-ray bucky are covered with disposable surgical sheet

All on site employees were provided with 3-ply face masks with ear-loop in addition to a face shield which was readily available to all technicians and radiologists for use during direct patient care. Hand sanitizers were available throughout the radiology department especially in areas where COVID-19 patients were imaged. Employees were asked to carry alcohol spray bottles for hand hygiene if frequent hand washing was not feasible. Full personal protective equipment (PPE) (including N95 respirators, long-sleeved disposable fluid repellent gown, and gloves) were provided for staff in close or direct contact with patients (Fig. 2). Mossa-Basha et al. from the University of Washington, school of medicine described precautionary measures such as the use of N95 filtering face piece respirators, powered air-purifying respirators (PAPR), and HEPA filtration systems to increase air exchange in the imaging suits except for MRI [8].

The infection control team in our hospital arranged tutorials to technologists and radiologists teaching them methods of donning and doffing of PPE and the measures that should be taken to disinfect the imaging room in between patients’ scans. In addition, medical engineers provided support for the best practices to disinfect the different scanners. Due to the travel ban and the interrupted medical supply chain, there were PPE shortage in a very brief short times which was quickly addressed by the institution. Upon following these measures, low infection rate without recorded morbidity among our faculty and staff was achieved.

#### Screening and dealing with exposed or sick employees

The temperature of all on-site radiology employees was measured daily upon hospital arrival. PCR screening was performed to all employees who were in contacts to proven cases. Symptomatic individuals with positive tests were quarantined in transmission-based precautions until at least two consecutive respiratory specimens become negative with improvement of respiratory symptoms (mainly cough and/or shortness of breath) and resolution of fever without the use of fever-reducing medications (test-based strategy). Asymptomatic individuals with positive PCR results would also be in transmission-based precautions until having negative test results from at least two consecutive respiratory specimens collected within 24 h (test-based strategy) [13].

With the increase in number of COVID-19 positive cases, employees were isolated only if they developed upper respiratory tract symptoms. Symptom-based strategy was followed for symptomatic individuals who would remain in transmission-based precautions or isolation until at least 10 days have passed since the symptom(s) onset provided that at least 24 h (initially 3 days) have passed since symptoms have been resolved, that is, resolution of fever without the use of fever-reducing medications with improvement in respiratory symptoms (cough and/or shortness of breath) [14].

On the other hand, in University of Washington School of Medicine, asymptomatic COVID-19 exposed employee was required to return to work and PCR tests were not performed; however, PCR tests were performed only for employee exhibiting upper respiratory tract symptoms [8].

#### Radiology staff wellness

In difficult times, caring for the mental and social wellbeing is as important as physical health. Although we are physically apart, we had to be connected, communicating. Socialization promotes health and safety feeling [15]. We actively encouraged all employees in the radiology department to interact through the various social media network. We launched a Radiology family WhatsApp group in which faculty members would send daily hopeful, cheerful, and encouraging messages to all members. Also, any achievements were recognized and mentioned in that group. In addition, we launched a financial support WhatsApp group. Monetary contributions were donated pay the support staff and families who were financially affected by the pandemic. Furthermore, monthly department council meetings have continued using Cisco’s WebEx video conferencing platform. Similarly, Morale-building activities have been promoted in other universities globally. For instance, University of Cincinnati offered virtual fitness challenges. Similarly, university of Wisconsin offered virtual yoga sessions and encouraged one-on-one communication especially for vulnerable staff, while New York University organized regular virtual coffee hours [5].

### Second domain: radiological examinations and patients’ safety

After discussing with the referring physicians, it was decided that elective imaging studies or procedures were to be postponed in order to protect vulnerable patients and staff from infection and promote physical distancing. In addition, through phone calls, outpatients who had scheduled elective procedures were notified to reschedule their examination; however, imaging procedures for critically ill and oncology patients were not rescheduled.

Similar measures were followed in University of Washington School of Medicine and Department of Radiology and Biomedical Imaging, University of California-San Francisco as well as Department of Radiology, Sant’Andrea University Hospital La Sapienza [8, 16, 17].

In order to implement precautionary measures on patients’ arrival, it was decided that the radiology request has to clearly indicate whether the patient was a confirmed or suspected COVID-19 case.

**Fig. 3:**
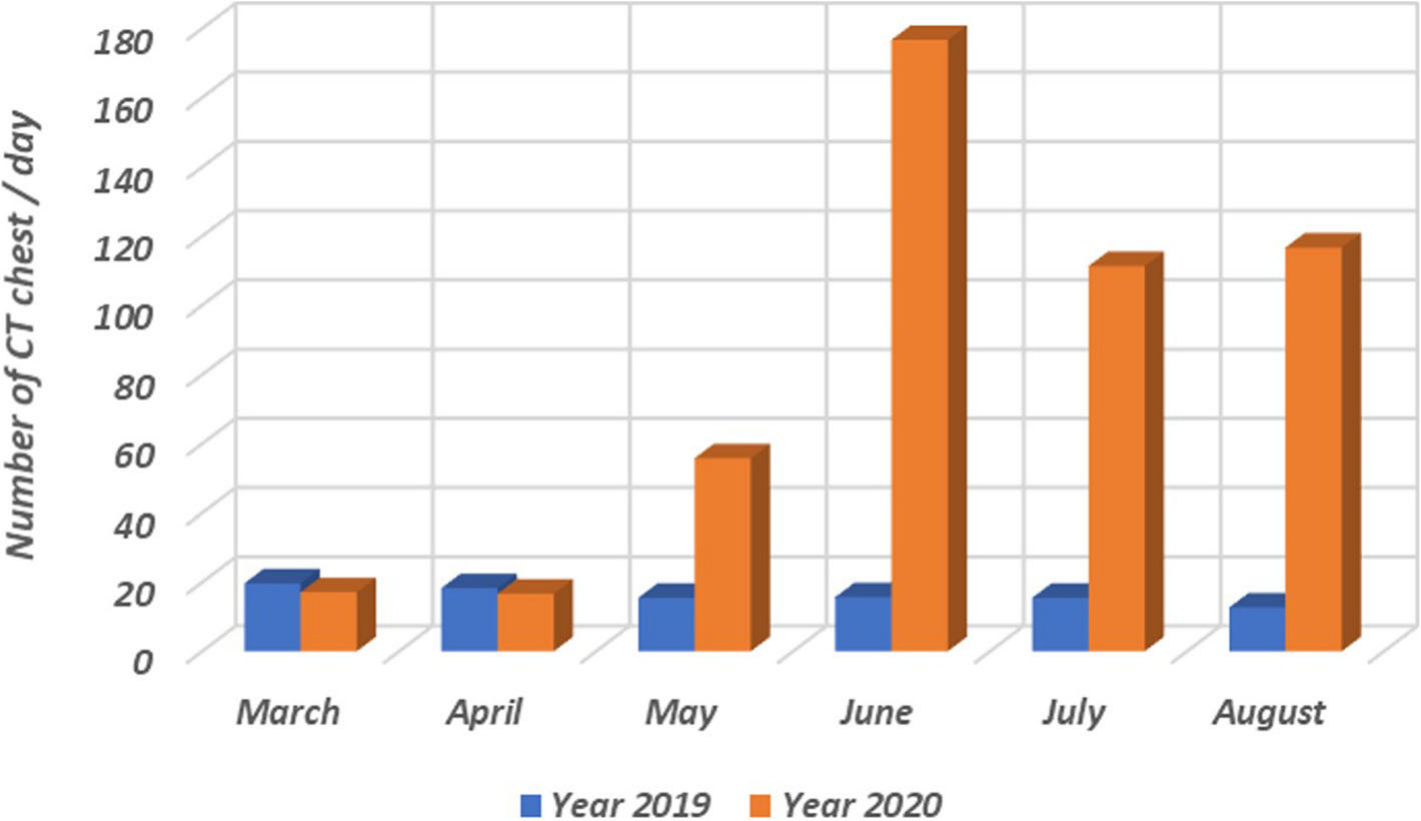
Bar graph demonstrating the average number of chest CT performed per day from March to August in 2020 compared to the number of examinations in the same period last year

**Fig. 4:**
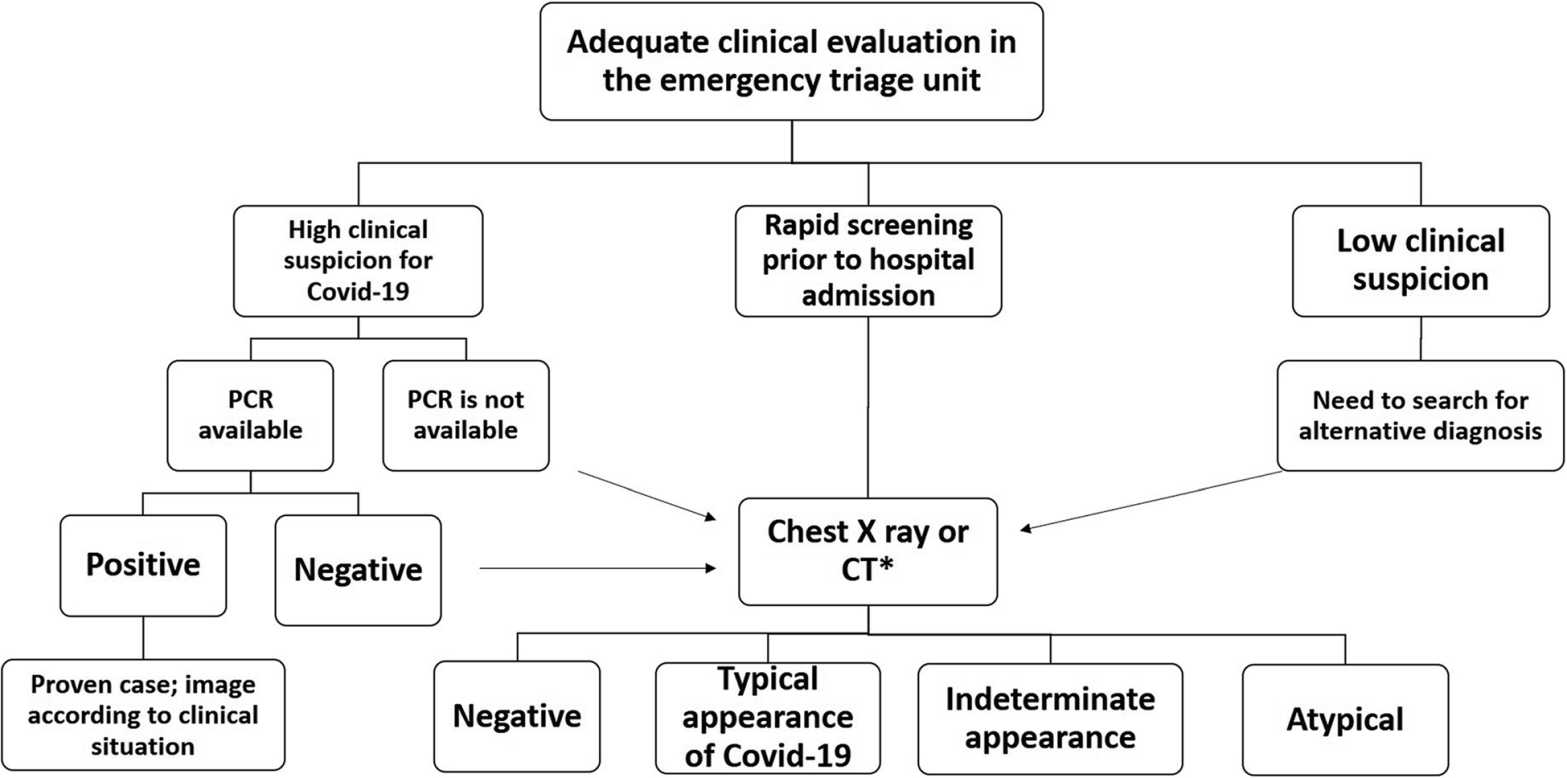
Recommended diagnostic algorithm for suspected COVID-19 cases presented in our emergency triage unit. Reporting of Chest CT for COVID-19 (as negative for pneumonia, typical, indeterminate, or atypical for COVID-19) was adopted from Simpson et al, 2020 [19]

Imaging is not usually indicated for suspected COVID-19 cases with mild clinical features unless they are at risk of disease progression. However, in resource-constrained environment where COVID-19 testing was not available, imaging was indicated for suspected patients presenting with moderate to severe symptoms and signs suggesting high probability of the disease [18]. Moreover, being highly sensitive and having short test-to-result time interval compared to PCR, CT referrals have tremendously increased (Fig. 3) in our department. CT may demonstrate typical features for COVID-19 or suggests an alternative diagnosis, necessitating rapid triage for those patients. After confirmation of COVID-19, further imaging is indicated if the imaging would alter the clinical management (such as stroke, or suspected pulmonary thromboembolism, or suspected intracranial hemorrhage) [18, 19] (Fig. 4).

In our department, one CT machine (out of five available CT scanners in our institution), one digital radiography, and one ultrasound machine were dedicated to image COVID 19 cases. Over time and with the exponential increase of the number of positive cases, a second CT scanner was dedicated to cover the increasing demands for CT chest exams. Portable chest X-ray and ultrasonography were the main imaging modalities in the ICU for critically ill patients. Similarly, dedicated imaging rooms were used for patients with confirmed or suspected cases of COVID-19 at department of diagnostic Imaging in the Hospital for Sick Children, in University Ave. In addition, rapid isolation rooms were allocated for a secondary screening [20].

Recently, a dedicated afternoon emergency outpatient clinic was created to serve suspected COVID 19 patients with availability of walk-in for chest CT scans, drive through labs through the medical research center for immediate care by a team of consultant radiologists, clinical pathologists, and pulmonologists.

In addition to radiography, CT, and ultrasound as diagnostic modalities, the workload in interventional radiology was reduced, while organ or life-saving procedures were maintained. Many central lines were placed for patients with COVID-19 admitted to ICU with interventional radiologists wearing PPE. The interventional radiology suit was deeply cleaned and disinfected after performing procedures for confirmed or suspected COVID-19 patients. Similar measures were followed in the Department of Radiology, Sant’ Andrea University Hospital La Sapienza [16].

### Third domain: education

#### Increase awareness of COVID-19 imaging features

At the institutional level, video awareness campaigns were initiated across the university. The “Treat and Teach” telemedicine initiative which launched in 2016 to reach remote and under privileged areas had become more on demand when the pandemic started.

At the radiology department level, awareness of the radiological features and patterns of COVID-19 on chest X-ray and CT images was our main goal. Distant teaching strategy was desperately needed to educate all radiologists (from residents to professors) about the specific radiological features of COVID-19 in order to be consistent in our reporting language when we discuss suspected cases with the clinicians or dictate our final reports. The thoracic imaging radiologists and pediatric radiologists delivered online webinars featuring detailed information about the Chest X ray and CT findings of COVID-19 infection in adults and children. While, we adopted the structured reporting for chest CT endorsed by the Radiological Society of North America (RSNA) (Fig. 4), Society of Thoracic Radiology, and the American College of Radiology [19], Covid-19 Reporting and Data system (CO-RAD) has been applied in other institutions when reporting suspected cases of COVID_19 [21]. On the other hand, the department of radiology in New York University discouraged the use of the term “COVID-19” within their radiology reports due to the great overlap between COVID-19 imaging findings and other diagnoses (atypical pneumonia, drug reaction, organizing pneumonia). Imaging helps provide baseline estimation of the extent of pulmonary affection and to indicate alternative diagnoses [7].

Radiologists were also encouraged to attend various webinars about COVID-19 hosted by Egyptian society of radiology, ESR (ESR-Connect), and RSNA [22–24]. Furthermore, images of X-rays and CT images of confirmed COVID-19 cases were privately shared on a newly launched emergency radiology WhatsApp group for networking while preserving patient confidentiality. Most of the recent publications discussing COVID-19 imaging features and management plans were also shared among the radiology faculty members.

#### Undergraduate and postgraduate medical education

COVID-19 pandemic has been an unprecedented challenge to education in our institution. It came at a critical time when the faculty of medicine was at the end of the semester and starting to arrange for spring exams. Teachings and assessments via the standard didactic face to face education, in class practical and clinical objective structured clinical exams had suddenly, but briefly, come to a halt to brainstorm until the state and institutional decisions were made. Medical students were asked to rely on distant education via online access of educational material, and online formative and summative assessments. Furthermore, the traditional final exams were replaced with research paper writing on the topics which has been taught throughout the term.

Teaching and examinations (including the written, practical, and clinical exams) via the internet were a major challenge. In a third-world setting, not all students had an active official university email to access online learning management system. This was quickly addressed by posting invitations on social media (such as the faculty of medicine and radiology Facebook pages) to all undergraduate students to activate their official emails in order to access educational materials and assessments during the crisis.

Some faculty and staff were not used to using their official university emails. These faculty members were also encouraged to activate their official emails, create online accounts on Moodle platform (a learning management system), and to prepare and upload their educational materials in the system to be accessed by medical students. The pandemic helped us assess the reality of digitalization as a higher education institution and the need to improve digital skills to offer appropriate distance learning. Compared to other developed countries, there is evidence that the Nordic countries—Finland, Sweden, Denmark, Estonia, Ireland, and the Netherlands—have significantly higher digital readiness than most of the Central and Southern European countries [25] and assumingly higher digital gap when compared to developing countries.

Regarding postgraduate education, the regular face to face lectures were also postponed. All masters and doctorate degree were encouraged to watch online educational material that were uploaded by the radiology faculty and attend educational webinars on Web Ex and Zoom platforms which were taught by the radiology faculty besides attending free webinars on various radiology topics offered through the American and European societies of pediatric, neuroradiology, head and neck imaging in addition to the Royal college of radiology, Egyptian, and Middle Eastern radiology societies [26–28]. Moreover, there were more new group discussions on a weekly basis using Telegram, WhatsApp, Zoom, and Facebook to cover various imaging topics, and case spot diagnosis of different imaging modalities. Distant learning using the visual thinking strategy created a fun and strong collaborative community that perfectly suited our radiologists in such difficult pandemic time.

For postgraduate exams, the radiology staff members from each of the different subspecialties created a radiology MCQ bank once the pandemic started. The multiple choice MCQ exam format was chosen over long essay format in order to shorten the exam time, grade the answer sheets automatically through scanner scoring machines rather than asking the staff member to travel to the school of medicine for grading, thus breaking the mandatory norm to adhere to physical distancing. Moreover, masters and doctorate level thesis defense were allowed to be conducted virtually using Cisco WebEx video conference platform to avoid delaying the students from entering their respective postgraduate exams.

Through social media and WhatsApp, focus groups were asked about their experience and future expectations in terms of future teaching strategies. Many radiology staff members expressed that COVID-19 was a great opportunity and experience that taught us how to shift our usual clinical practice, teaching methods, and opened new convenient channels to cope with the pandemic.

### Fourth domain: research

Research at the Radiology department Ain Shams university is centered on different radiology subspecialties ( pediatric imaging, cardiovascular, thoracic imaging, neuroradiology, head and neck, breast imaging, gynecology and obstetrics, musculoskeletal, interventional radiology, oncology) and involves imaging research in various modalities (radiography, ultrasound, CT, MRI, and nuclear medicine). Research programs are mainly the outcome of master and doctorate thesis in the radiology department in collaboration with other clinical disciplines. Once the pandemic started, research activities were markedly reduced aiming for minimizing the spread of infection among researchers, employees, and community. New research was focused on novel COVID-19 aiming for understanding of the imaging features and management of the newly emerging virus.

Researchers were encouraged to work remotely from home and were instructed to enhance their writing capabilities through attending online classes and workshops. Also, the research team leaders encouraged team members to continue their activities in literature search, data analysis, manuscripts, and grant writing. Other retrospective research projects which deal with retrieving archived files were also encouraged.

Similarly, the collective experiences of western academic radiology research centers were recently documented. For instance, all human research subject studies involving personal interactions were halted in university of Cincinnati. Non-essential research was discontinued in university of Wisconsin. In Johns Hopkins University, only COVID-19-related research was allowed. A “Research Slowdown Planning tool” and “Ramp-Down Non-Essential Laboratory Research” were developed in Emory University to identify functions related to research continuity and to help guide decisions regarding remote working from home [5].

### Fifth domain: finances and donations

The financial impact of COVID-19 is well known. The decrease of workload tremendously affected financially the radiology departments globally. Several approaches have been taken worldwide to decrease the expenses and overcome the financial crisis [29]. Besides the government’s financial allocation to the whole institution, significant donations from our radiologists, medical students, and the community were received to purchase PPEs, a mobile X-ray for the pediatric isolation unit, a portable ultrasound machine, and three digital radiography systems in the form of flat panel and their workstation. On comparison with developed institutions, the department of radiology in university of Alabama received financial support from hospital administration was important for hardware virtual private network (VPN) and display monitors for implementation of home PACS [12]. While university of Washington Medical Center Northwest eliminated a tele-radiology service that was providing overnight coverage, our radiology staff offered working remotely at day and night shifts (24/7) to face the increased demand of rapid reporting especially for the requested chest CT. To be more crisis resilient, University of Washington on the other hand, structured long-term revenue diversification plans to replenish financial reserves [29].

## Conclusion

COVID-19 pandemic was not only a challenge to the radiology department in developing country but also to radiology departments in developed countries. It was a great opportunity for building resilience to adapt to change. Covid-19 pandemic has opened the door for changes in radiology department policies and practices. It also has accelerated innovation in teaching and clinical practice through implementation of online learning tools and activation of home PACS. The crisis also drew special attention to the role of governmental support and donations. The challenges we faced during the pandemic taught us to reimagine new adaptable policies and practices that may likely persist after passing of the pandemic and in case of future surges and crisis.

## Data Availability

All data provided are publicly known and available and are included in this published article.

## Abbreviations

Covid-19: Corona virus 19 virus
Co-RAD: Covid-19 Reporting and Data system
ESR: European Society of Radiology
MCQ: Multiple choice questions
PPE: Personal protective equipment
PCR: Polymerase chain reaction
RSNA: Radiologic society of North America
